# Whole genome comparison of *Aspergillus flavus* L-morphotype strain NRRL 3357 (type) and S-morphotype strain AF70

**DOI:** 10.1371/journal.pone.0199169

**Published:** 2018-07-02

**Authors:** Matthew K. Gilbert, Brian M. Mack, Geromy G. Moore, Darlene L. Downey, Matthew D. Lebar, Vinita Joardar, Liliana Losada, JiuJiang Yu, William C. Nierman, Deepak Bhatnagar

**Affiliations:** 1 Food and Feed Safety Unit, USDA, New Orleans, Louisiana, United States of America; 2 The J. Craig Venter Institute, Rockville, Maryland, United States of America; 3 Food Quality Laboratory, USDA, Beltsville, Maryland, United States of America; University of Nebraska-Lincoln, UNITED STATES

## Abstract

*Aspergillus flavus* is a saprophytic fungus that infects corn, peanuts, tree nuts and other agriculturally important crops. Once the crop is infected the fungus has the potential to secrete one or more mycotoxins, the most carcinogenic of which is aflatoxin. Aflatoxin contaminated crops are deemed unfit for human or animal consumption, which results in both food and economic losses. Within *A*. *flavus*, two morphotypes exist: the S strains (small sclerotia) and L strains (large sclerotia). Significant morphological and physiological differences exist between the two morphotypes. For example, the S-morphotypes produces sclerotia that are smaller (< 400 μm), greater in quantity, and contain higher concentrations of aflatoxin than the L-morphotypes (>400 μm). The morphotypes also differ in pigmentation, pH homeostasis in culture and the number of spores produced. Here we report the first full genome sequence of an *A*. *flavus* S morphotype, strain AF70. We provide a comprehensive comparison of the *A*. *flavus* S-morphotype genome sequence with a previously sequenced genome of an L-morphotype strain (NRRL 3357), including an in-depth analysis of secondary metabolic clusters and the identification SNPs within their aflatoxin gene clusters.

## Introduction

*Aspergillus flavus* is an agriculturally significant fungus, whose pathogenesis can occur in either field or post-harvest conditions [1]. This fungus may also secrete one or more toxic secondary metabolites, termed mycotoxins, the most carcinogenic of which is aflatoxin [2]. Aflatoxin exists naturally in several forms depending on the fungal strain, the most potent of which is aflatoxin B_1_ (AFB_1_)[3]. Aflatoxin contamination of food and feed results in commodities that are unfit for consumption, which equates to millions of dollars in economic losses annually [3–6].

Within species *A*. *flavus*, two morphological groups (i.e. morphotypes) have been characterized based on sclerotia production: the S strains (small sclerotia) and the L strains (large sclerotia) [7]. The S morphotype typically produces sclerotia less than 400 μm in diameter and produce higher quantities of AFB_1_ in culture than the L morphotype. These also differ in pigmentation, pH homeostasis in culture, and the number of sclerotia and spores produced [7–10]. Previous sequence analysis of three genes from a collection of *A*. *flavus* strains indicated that two distinct phylogenetic groups (groups I and II) exist within the species, however this does not allow the complete discernment between toxigenic/atoxigenic, or between S- and L-morphotypes. Group I consists of both S- and L-strains that produce AFB_1_, while group II is comprised of S-strains that produce AFB_1_ and/or G-aflatoxins (AFG) [8, 11] (although producers of the AFG metabolite appear to be found in Group I as well). Because significant morphological and physiological differences exist between the two morphotypes, a comparison of the two is useful for elucidating mechanisms of virulence, toxin production and development.

Both S and L strains of *A*. *flavus* are pathogenic to agricultural crops. Horn *et al*. [12] found, in a survey of peanut fields throughout the southern United States, that nearly all S strains and approximately 70% of L strains produce both AFB_1_ and another toxic metabolite, cyclopiazonic acid (CPA). A later study by Abbas *et al*. [9], in the Mississippi Delta, reported that L strains exhibited much higher virulence since they were far more prevalent in corn, peanut, rice and soil samples than S strains, however all crops were susceptible to AFB_1_ contamination by at least one morphological type.

NRRL 3357 (ATCC 200026, GenBank assembly accession: GCA_000006275.2) is an *A*. *flavus* L strain morphotype that has been developed as a model organism over many years, and across multiple studies, evaluating secondary metabolite production [13–15], genome profiling [16, 17], proteome functional analysis [18], contamination of crop supplies [19, 20], the development of biological control agents [17], and many others. The sequencing of strain NRRL 3357 was the first fully sequenced *A*. *flavus* genome, initially reported on by Payne *et al*. [21], and subsequently re-sequenced to produce a less fragmented genome by Nierman *et al*. [22]. The S strain sequenced for this study (AF70) was originally isolated using a dicloran-amended media from soil obtained in a Pima Cotton field in Yuma Valley, AZ, USA, and first characterized by Cotty *et al*. in 1989. It produces very high AFB_1_ levels, especially during infection of maize kernels [7]. Strain AF70 has been used for the characterization of fungal development and growth [23, 24], virulence of crops [25], secondary metabolite production [15], oxidative stress [26], genome profiling [27] and numerous other studies.

Here we report on morphological differences and vegetative compatibility, and provide a genome-wide assessment of the important differences, between strains AF70 and NRRL 3357. Further, we describe the sequencing and methodology used for the annotation of strain AF70, which utilized RNA-seq reads from available experiments to train gene prediction algorithms. Our analysis of the secondary metabolic gene clusters revealed significant differences in the metabolic profiles of these strains, and we identify key single nucleotide polymorphisms (SNPs) in their respective aflatoxin gene clusters.

## Materials and methods

### Strains and growth conditions

*Aspergillus flavus* AF70 (ATCC 200026) [7] was sequenced using the techniques described below. Genomic comparisons were made to *A*. *flavus* strain NRRL 3357 [22]. For imaging, determining spore production, sclerotia diameter, and AFB_1_ analysis, fresh spores from both strains were first produced by plating stocks on V8 agar plates (10% V8 juice, 3% ammonium sulfate, 1% uracil, pH 5.2). For AFB_1_ analysis, Yeast-Glucose-Trace Element (YGT) medium was supplemented with 6.0 g/L casamino acids and 1 ml/mg trace element solution as previously described [28–30]. The YGT consisted of 0.5% yeast extract, 2% glucose, 20 g agar, and 1 ml of trace element solution per liter of medium [28]. Selection media used in vegetative compatibility group (VCG) assays consisted of amended Czapek-dox (CZ; Difco, BD)), Mutation Induction Media (MIT), Nitrate, Nitrite or Hypoxanthine media [31]. The amended CZ included 35 g of CZ broth, 25 g KClO_3_, 10 ml of 5 mg/ml Rose Bengal stock solution (Sigma-Aldrich, St. Louis, MO) and 2% Bacto agar (Sigma-Aldrich) per liter, pH 7.2. MIT media consisted of 35 g CZ broth, 15 g KClO_3_ and 2% Bacto agar per liter with a final pH of 6.5. Nitrate medium consisted of (per L) 35g CZ, 2% Bacto Agar, Nitrite media consisted of (per L) 50g Sucrose, 10g KH_2_PO_4_, 2g MgSO_4_-7H_2_O, 1 ml micronutrients, 10mM NaNO_2_, and 2% Bacto Agar. Hypoxanthine media was identical to nitrite media except 100 mg/L hypoxanthine replaced NaNO_2_.

### Phenotypic comparison of *A*. *flavus* morphotypes

For obtaining images of fungal phenotype, 2x10^5^ fresh spores from strains AF70 and NRRL 3357 were spot plated on YGT agar plates and grown for 6 days at 30 °C. Stereoscope images were taken on a Zeiss Stereoscope Discovery.v20 using an Axiocam MRc5 digital camera system (Zeiss, Oberkochen, Germany). To determine spore production, 1x10^6^ fresh spores were point inoculated on YGT and V8 media plates, and grown for 9 days at 30 °C in 12 hr light/dark cycles. Three representative plugs were removed from each plate and added to 500 μl 0.02% Triton-X 100 (TTX-100). A hemocytometer (Hausser Scientific, Horsham, PA) was used for counting spores, and in every case >100 spores were counted. Three independent plates for each strain and medium were used.

To determine sclerotium diameter, 2x10^5^ fresh spores from each strain were point inoculated on YGT plates. After 6 days of growth on YGT agar, sclerotia were harvested with 0.01% TTX-100 and rinsed onto filter paper (Whatman #4, Whatman Ltd.). Three independent plates for each strain were used. Sclerotium diameter was measured by averaging two cross-measurements. Measurements were conducted using Axiovision 4.8 image analysis software (Zeiss).

To determine if both morphotype representatives are of the same VCG, the method of Horn *et al*. [32] was used with some modification. Spores were inoculated onto 0.5X V8 plates. After 5 to 7 days of growth, five agar plugs containing spores were cut and put into sterilized glass vials containing 2.5 ml deionized water. Twenty microliters of each spore suspension was then inoculated onto the center of multiple plates containing a single type of selection medium. Between days 5–14, mutations were identified as a cloudy, thick growth that outgrew the wild-type. Observed mutants were then transferred to MIT agar plates for further selection. After approximately 3 days of growth, 1 plug from the outer growth was then transferred to a nitrogen-selecting medium, where vegetative compatibility was determined by pairing complementary mutants (*nia-D*, *nir-A* or *cnx*).

### Aflatoxin extraction and ultra-performance liquid chromatography (UPLC) analysis

After growth of mycelia on solid supplemented YGT media, 220 ml of 2:1 acetone:water was added and shaken vigorously for 16 hours, then allowed to separate. The supernatant was extracted with 150 ml methylene chloride, which was then evaporated under filtered nitrogen gas. Each dried sample extract was resuspended in 1 ml acetonitrile, then transferred to a 2 ml, 0.45 μm nylon filter centrifuge tube (Spin-X; Corning Inc., Corning, NY) and centrifuged at 14000 rpm for 1 min. Injections (1 μl) of filtered extract were analyzed by UPLC. AFB_1_ and AFB_2_analysis was performed with modifications to a procedure previously described [33]. UPLC analyses were performed with a Waters Acquity H-Class System combined with an Acquity fluorescence (FLR) detector (Waters, Milford, MA). Sample extract (1 μl) was injected for separation through an Acquity BEH C18 column (1.7 um, 2.1 x 50 mm) at 30 °C. Run time was 3 min with an elution flow rate of 0.4 ml/min and isocratic mobile phase consisting of methanol:water (40:60). AFB_1_ detection wavelength was 365 nm (excitation) and 440 nm (emission) and retention time was 2.1 min. A calibration curve with high linearity (R^2^ = 0.9993) was constructed for AFB_1_ from a series of diluted standards (Sigma-Aldrich). Statistical analysis conducted using Graphpad Prism 7 (Graphpad Software Inc).

### Sequencing and assembly of the *A*. *flavus* AF70 genome

DNA was extracted from 10 g of *A*. *flavus* AF70 mycelia harvested after 24 h growth in PDB (Becton, Dickinson and Co., Franklin Lakes, NJ) and continuous shaking at 30 °C. Briefly, mycelia were ground to a fine powder in liquid N_2_, then subjected to CTAB extraction (1% CTAB [mixed alkyltrimethyl-ammonium bromide], 200mM Tris-HCl pH8.0, 0.8M NaCl, 1% B-mercaptoethanol, 10mM EDTA), followed by Proteinase K (500ug/ml) digestion, two rounds of phenol:chloroform extraction and ethanol precipitation [34]. Long strands of visible DNA were removed using a glass rod, and shipped frozen to J. Craig Venter Institute, Bethesda, MD. The sample quality was assayed using an agarose gel and an Agilent 2100 bioanalyzer (Santa Clara, CA). Sequencing of strain AF70 was performed using single end 100 bp reads in the Illumina GA-II (Illumina, San Diego, CA). A total of 20,255,792 reads were obtained equivalent to roughly 70X sequence coverage. The assembly was done using CLC Genomics Workbench de novo assembler (v4.3). Raw Reads are available at the sequence read archive, SRA accession: SRP131888 (www.ncbi.nih.gov/sra).

### Genome annotation

Gene predictions were performed using the MAKER pipeline (version 2.31.4) [35]. First, repetitive elements were identified in the initial AF70 genome sequence by MAKER using the RepeatMasker [36] algorithm in conjunction with the RepBase [37] repeat library for fungi and MAKER’s built-in repeat database. To train the gene prediction algorithm Augustus [38], Trinity was used to assemble a transcriptome from in-house generated RNA-seq experiments for *A*. *flavus* AF70 [23]. MAKER was then run using the assembled transcriptome, the predicted proteins from *A*. *oryzae*, *A*. *flavus* NRRL 3357, and *A*. *nidulans* (with the “protein2genome” and “est2genome” options set to 1), and fungal protein sequences from the SWISS-Pro database to develop a set of predicted genes. These genes were filtered using maker2zzf based on how well they fit the EST evidence, and narrowed down to about 1,200 genes which were then used to train the first iteration of Augustus. MAKER then produced *ab initio* gene predictions from the repeat-masked AF70 genomic sequence using the GeneMark [39] and trained Augustus. Gene prediction was also performed on the AF70 sequence using the PASA pipeline [40]. These sets of predictions were included in subsequent runs of MAKER through the “pred_gff” option. Augustus was retrained using results from the second run of MAKER, and MAKER was run a third time. All runs of MAKER were performed with the “single_exon” set to 1 and “correct_est_fusion” set to 1. Gene predictions that had no overlapping EST or protein homology evidence were included in the final set of predictions if they were found to have an InterPro domain when examined using InterProScan. The final annotations were manually edited using WebApollo (version 2013-11-22). The sequence and annotation file was uploaded to NCBI under Genbank Assembly Accession GCA_000952835.1. The genome sequence and predicted proteins for *A*. *oryzae* and *A*. *nidulans* were obtained from AspGD (www.aspergillusgenome.org, accessed 9/15/14). The sequence for NRRL 3357 was obtained from NCBI (accessed 4/1/14).

### Comparative analyses of *A*. *flavus* morphotypes

For construction of Venn Diagrams, coding sequences were aligned using blastn. Sequences with coverage > 50%, identity over 70% and an e-value < 1e-10 were considered orthologous. For Gene Ontology Enrichment Analysis, the R package GOSeq was used [41]. Construction of the phylogenetic tree involved orthologous proteins that were detected using Proteinortho [42], aligned with Muscle [43], and concatenated into a 1.1 Mb amino acid alignment using GBLOCKS [44]. The tree was produced using RAxML with *A*. *zonatus* as the outgroup taxon. The inset shows closely related species with expanded branch lengths. For secondary metabolic cluster prediction, the Antibiotics-Secondary Metabolite Analysis Shell (anti-SMASH) program was used [45]. Default parameters were used except for the incorporation of the ClusterFinder algorithm. Anti-SMASH is designed to predict 43 categories of gene clusters (e.g. Type 1–3 polyketide synthases [PKS], Non-ribosomal peptide-synthetases [Nrps], terpenes, etc.), thus it generally provides a relatively comprehensive list of cluster predictions. Examination of the clusters within the AF70 and NRRL 3357 genomes was undertaken using MultiGeneBlast [46] in tblastn mode. Each of the known eukaryotic cluster entries from the MIBiG database (accessed 7/1/17) [47] was used as a query against the AF70 and NRRL 3357 genomes. The entry with the most genes was chosen for clusters that had redundant entries. Variant analysis involved creating simulated short reads of the AF70 genome using bbmap [48], mapping those reads to the NRRL 3357 genome using BWA [49], calling variants using Freebayes [50], and annotating variants using SNPeff [51]. Although the total number of orthologous genes will be different than the method described above, the pipeline provides a more accurate estimation of SNP content and is in keeping with best practices [52].

We also utilized nearly 70 kb of genomic sequence from the aflatoxin gene clusters of AF70 (*A*. *flavus* S-morphotype) and NRRL 3357 (*A*. *flavus* L-morphotype) to compare the quantities and types of single nucleotide polymorphisms (SNPs) that differentiated their biosynthetic pathways. The aflatoxin cluster sequences for both strains were acquired from the NCBI database and uploaded as FASTA files to the sequence analysis program Sequencher (Gene Codes Corporation, Michigan). To accurately identify the start and end of each gene and intergenic region, within the aflatoxin cluster, we also added sequences from individual *A*. *flavus* cluster genes to the alignment. Once the cluster sequences were aligned, and the correct orientation of individual cluster genes was verified, the process of scrolling along the cluster sequences began. For each gene and intergenic region, observations were noted for the numbers of transition/transversion SNPs as well as deletions within each strain’s cluster sequence. Another comparison involved multi-locus sequence typing (MLST) for NRRL3357 and AF70. Alignments for each of six genomic regions were prepared using Sequencher and then exported as Nexus files: two aflatoxin cluster intergenic regions (*aflM/aflN* and *aflW/aflX*), and four non-cluster regions (*amdS*, *trpC*, *MAT1-1* and *mfs*). These loci have proven, in previous MLST studies for *A*. *flavus*, to accurately segregate individuals within a population in lieu of vegetative compatibility group (VCG) testing [53]. The Nexus files were then imported into a suite of nucleotide analysis programs (SNAP) that are used for population inferences [54]. Using one of the workbench programs, SNAP: Combine, we concatenated the six different alignments to make a single sequence alignment. Another program (SNAP: Map) was used to collapse the sequences into haplotypes using the parameters of recoded indels and excluded infinite sites violations. The resulting map file indicates whether or not each strain examined is considered an “individual” based on its haplotype designation [55].

## Results and discussion

### Morphological characterization of *A*. *flavus* morphotypes

**Fig 1:**
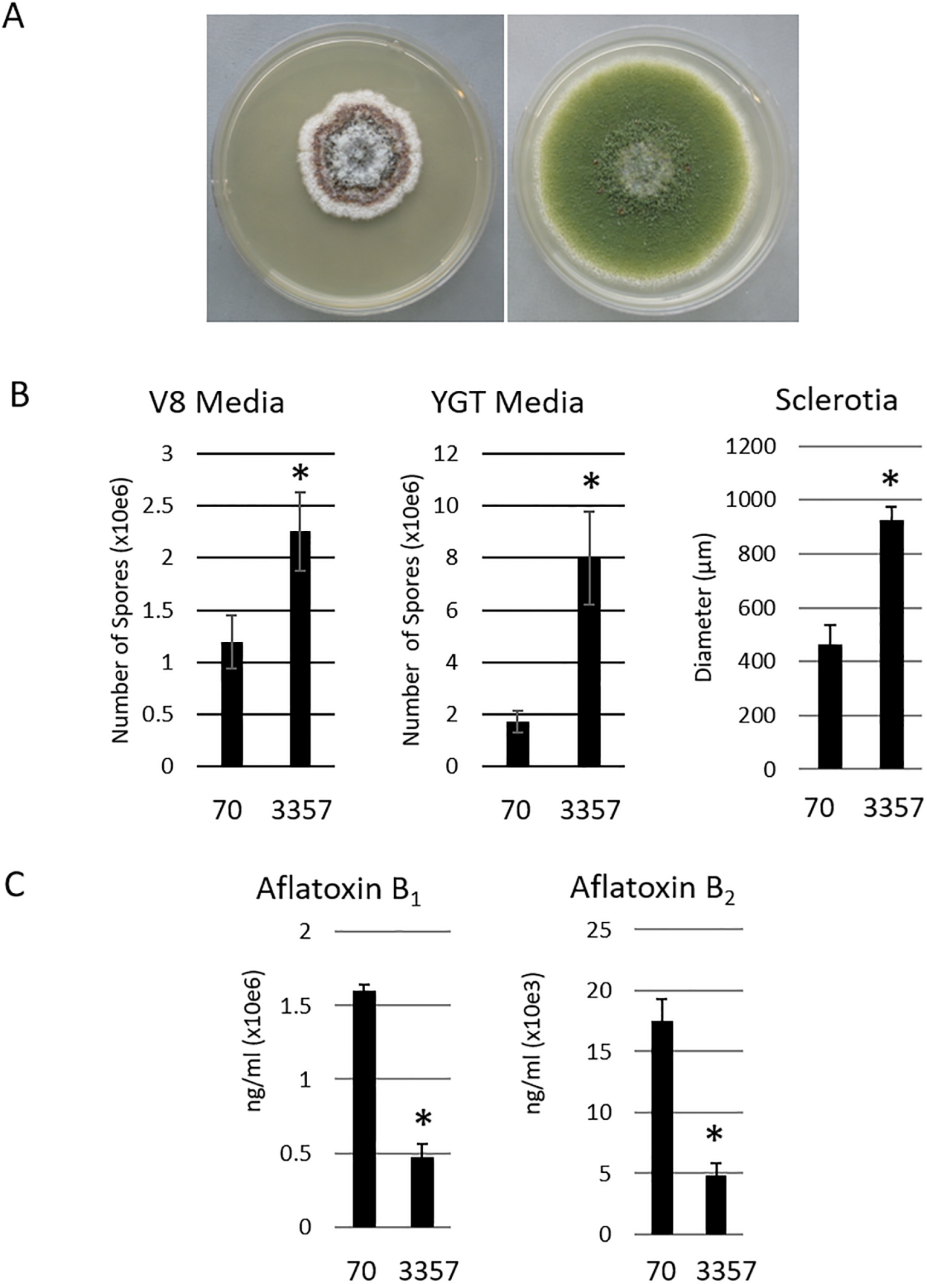
Phenotype comparisons for *A*. *flavus* strains AF70 and NRRL 3357. A) After 6 days growth on YGT media, S-morphotype AF70, left, demonstrated several rings of pigmentation correlating with production of numerous small sclerotia, with unpigmented hyphae in the outermost edges of growth. Conversely, L-morphotype NRRL 3357 maintains typical olive-green pigmentation with no sclerotia visible. B) Spore production on two media types, V8 and YGT showed NRRL 3357 consistently producing more spores. C) HPLC quantification of aflatoxin AFB production shows higher concentrations produced by S-morphotype AF70. Student’s t-test where *P* ≤ 0.05 indicated by asterisk (*).

The phenotypic differences exhibited between AF70 and NRRL 3357 are relevant for contextually analyzing a genomic comparison. While some characteristics have been reported previously [7], we wanted a direct and current comparison of their morphological characteristics, aflatoxin production and diameter of sclerotia. Although equal amounts of spore suspension from each strain were plated on YGT, the AF70 (S-morphotype) strain demonstrated slower growth, greater quantities of sclerotia, and a lack of olive-green conidia, compared to the NRRL 3357 L-morphotype strain (Fig 1A). AF70 contained significantly higher spore content (Fig 1B, left and center) and cross-sectional measurements of sclerotia indicated the average size of sclerotia was approximately 400 um, whereas the average diameter for NRRL 3357 sclerotia was just under 1000 um (Fig 1B, right). AFB_1_ and B_2_ levels were 4-fold higher and 3.5-fold higher, respectively, for S-morphotype AF70 than for L-morphotype NRRL 3357 (Fig 1C).

Collectively, our morphological comparisons of AF70 and NRRL 3357 support general observations relating to both morphotypes. It was also determined, through our VCG assays, that strains NRRL 3357 and AF70 are not vegetatively compatible (S1 Fig), which is consistent with previous findings [56].

### Genomic comparison of AF70 and NRRL 3357

**Fig 2:**
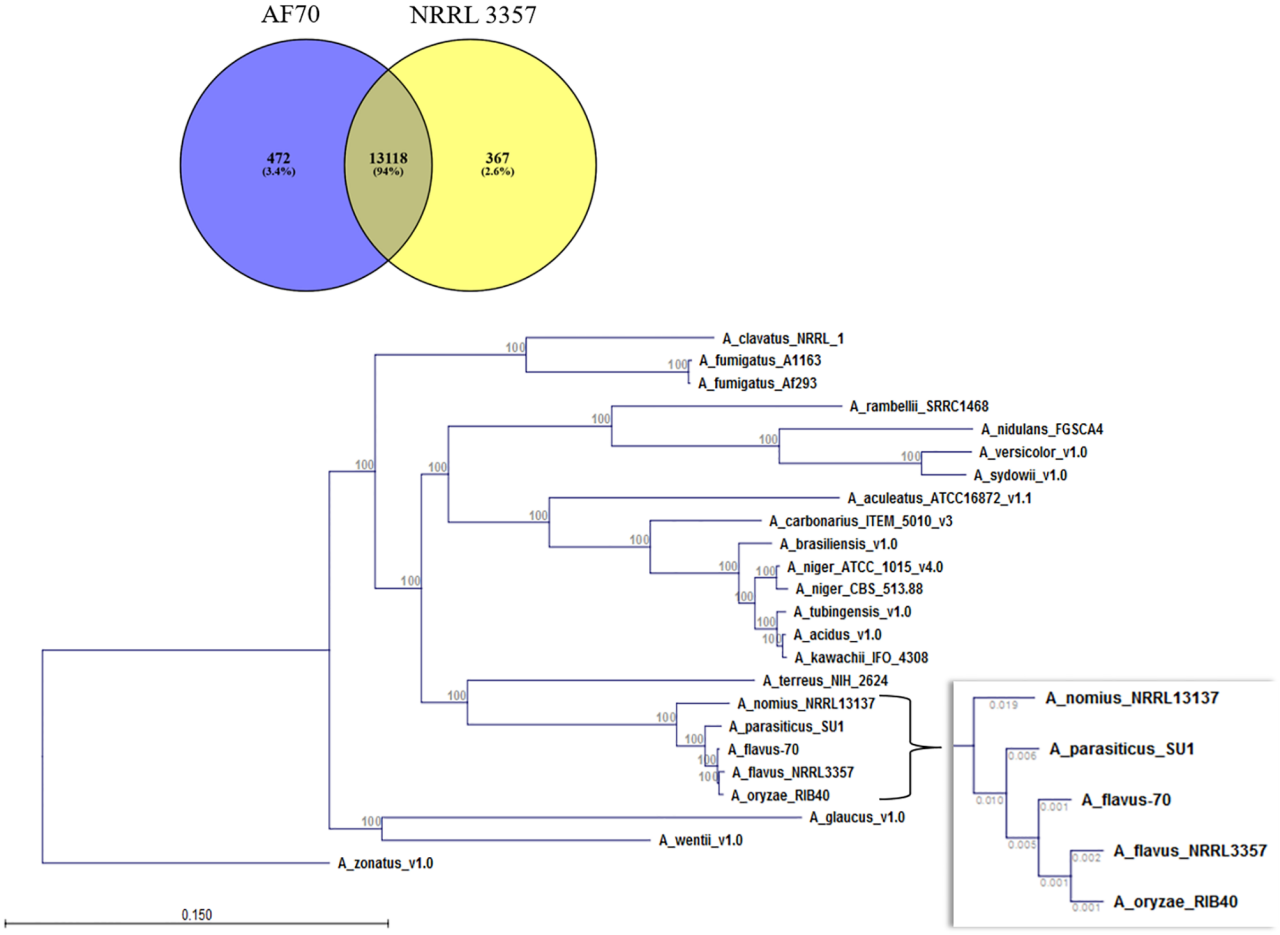
Genome comparisons of *A*. *flavus* strain AF70 and NRRL 3357. A) Number of genes identified as unique or shared between strains AF70 and NRRL 3357 (S and L strains, respectively) indicate 94% of genes are shared (orthologous). B) Protein sequences were compared and used to illustrate the hierarchal relationship between strains AF70, NRRL 3357 and closely related species, and indicate that NRRL 3357 (“A_flavus_NRRL3357”) is more closely related to *A*. *oryzae* (“A_oryzae_RIB40”) than *A*. *flavus* AF70 (“A_flavus 70”). Values on main tree indicate bootstrap values. Values on inset indicate branch length.

Of the approximately 13,500 genes predicted to exist in NRRL 3357 and AF70, 13,118 genes were predicted to be orthologous based on blastn results of the coding sequences versus the genome based on 70% identity, 50% query coverage, and an E value <1x10^-10^ (Fig 2). Also based on this criterion, 472 genes were found to be unique to AF70, while 367 genes were found to be unique to NRRL 3357. Using Proteinortho to determine the number of orthologues indicated 2,397 coding sequences unique to NRRL 3357 and 2,039 unique to AF70. However, it should be pointed out that differences in annotation could contribute significantly to this determination. Phylogenetic comparison including multiple *Aspergillus* species, and using concatenated amino acid sequence alignment, indicates NRRL 3357 shares highest sequence identity with *A*. *oryzae* (Fig 2B). Also observed was that both of the examined *A*. *flavus* morphotypes share a common ancestor. Gene enrichment analysis of the genes unique to each morphotype was conducted to determine if broad categories of genes are present/absent in either genome. Genes unique to strain AF70 were enriched in cytochrome P450 mono-oxygenases (GO:0016705 oxidoreductase activity, acting on paired donors, with incorporation or reduction of molecular oxygen) (Table 1 and S1 Table). Three of the identified genes are part of a unique gene cluster (AFLA70_220g001820, AFLA70_220g001850, AFLA70_220g001880).

**Table 1:** Gene Ontology categories of genes that are significantly enriched in strain AF70 and NRRL 3357.

Unique to Strain AF70			Unique to Strain NRRL 3357		
**Biological Processes**	**E value**	**Description**	**Biological Processes**	**E value**	**Description**
			GO:0009116	0.000	nucleoside metabolic process
GO:0055114	0.001	oxidation-reduction process	GO:0016998	0.000	cell wall macromolecule catabolic process
GO:0009116	0.001	nucleoside metabolic process	GO:0055114	0.001	oxidation-reduction process
GO:0015991	0.015	ATP hydrolysis coupled proton transport	GO:0034551	0.013	mitochondrial respiratory chain complex III assembly
GO:0038032	0.027	termination of G-protein coupled receptor signaling pathway	GO:0006098	0.044	pentose-phosphate shunt
**Molecular Function**	**E value**	**Description**	**Molecular Function**	**E value**	**Description**
GO:0016705	0.000	oxidoreductase activity, acting on paired donors, with incorporation or reduction of molecular oxygen	GO:0016491	0.000	oxidoreductase activity
GO:0020037	0.000	heme binding	GO:0004497	0.002	monooxygenase activity
GO:0005506	0.000	iron ion binding	GO:0004252	0.011	serine-type endopeptidase activity
GO:0016831	0.008	carboxy-lyase activity	GO:0003824	0.013	catalytic activity
GO:0015078	0.011	hydrogen ion transmembrane transporter activity	GO:0016614	0.015	oxidoreductase activity, acting on CH-OH group of donors
GO:0031177	0.020	phosphopantetheine binding	GO:0004616	0.016	phosphogluconate dehydrogenase (decarboxylating) activity
GO:0015299	0.022	solute:proton antiporter activity	GO:0050660	0.016	flavin adenine dinucleotide binding
GO:0016772	0.032	transferase activity, transferring phosphorus-containing groups	GO:0015124	0.020	allantoate transmembrane transporter activity
GO:0016740	0.032	transferase activity	GO:0045482	0.020	trichodiene synthase activity
GO:0008270	0.035	zinc ion binding	GO:0008762	0.024	UDP-N-acetylmuramate dehydrogenase activity
GO:0016491	0.039	oxidoreductase activity	GO:0016705	0.026	oxidoreductase activity, acting on paired donors, with incorporation or reduction of molecular oxygen
GO:0004497	0.049	monooxygenase activity	GO:0005506	0.031	iron ion binding
			GO:0020037	0.034	heme binding
			GO:0000293	0.039	ferric-chelate reductase activity
			GO:0005384	0.039	manganese ion transmembrane transporter activity
			GO:0042936	0.039	dipeptide transporter activity
			GO:0097079	0.039	selenite:proton symporter activity
			GO:0009055	0.049	electron carrier activity

### Secondary metabolic gene clusters in *A*. *flavus* AF70 and NRRL 3357

**Fig 3:**
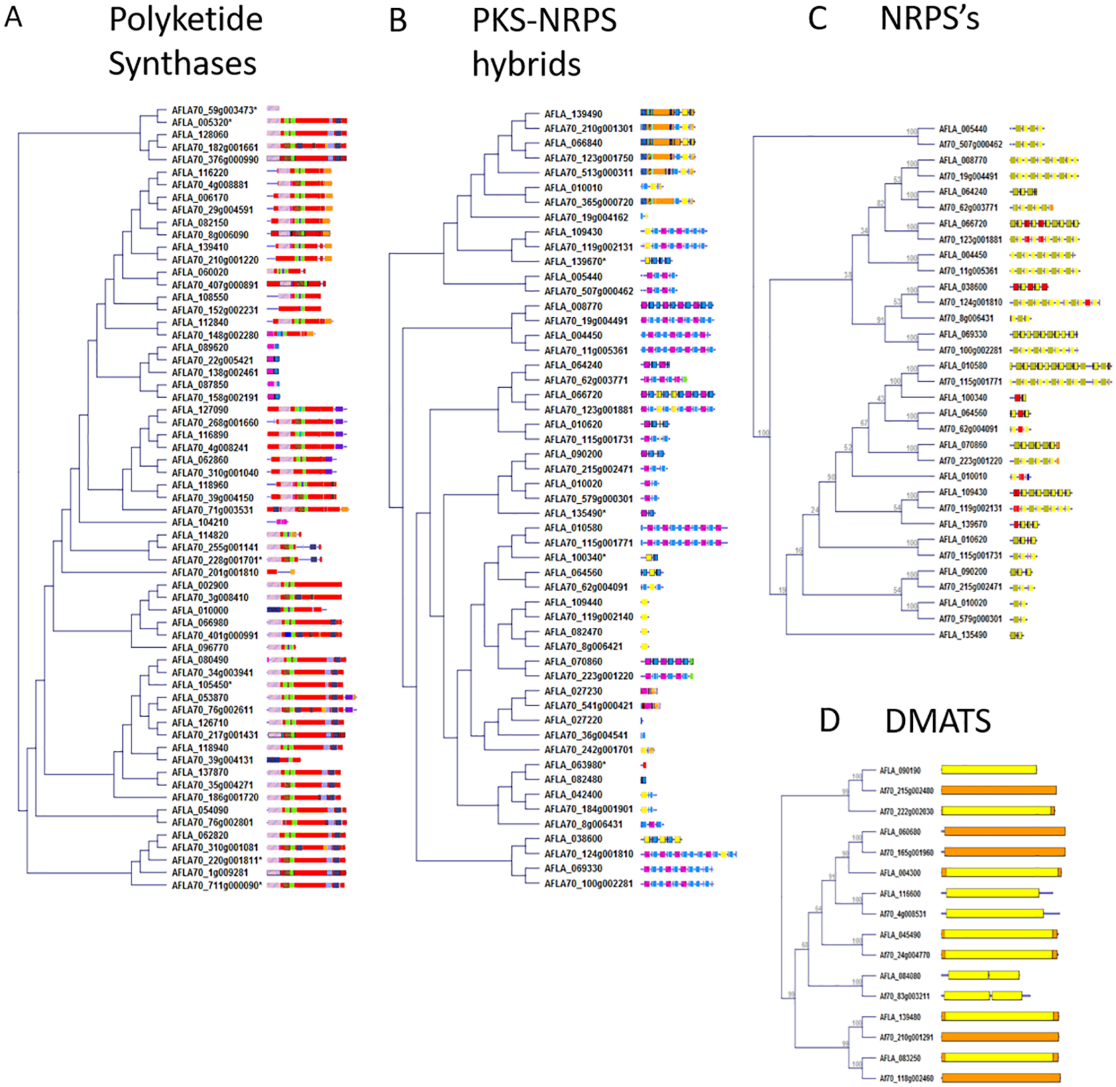
Secondary metabolic gene cluster backbone genes. The “backbone enzymes” of secondary metabolic gene clusters in *A*. *flavus* strains AF70 and NRRL 3357 (S- and L-morphotypes, respectively) were compared and clustered according to amino acid sequence similarity. The colored segments indicate domains identified by InterProScan. A) Polyketide synthases (PKSs), B) Polyketide synthase-nonribosomal peptide synthetase hybrids (PKS-NRPSs), C) nonribosomal peptide synthetases (NRPSs), and D) dimethylallyl tryptophan synthases (DMATs).

The metabolic clusters within *A*. *flavus* strains AF70 and NRRL 3357 were identified by antiSMASH, and alignment of each of the PKS, PKS-NRPS hybrid, NRPS and dimethylallyl tryptophan synthetase (DMAT) enzymes was conducted to illustrate the identity of the domains within each enzyme (Fig 3A–3C). The genomes of AF70 and NRRL 3357 were queried for known eukaryotic biosynthetic clusters using tblastn with MultiGeneBlast, which searches for each gene individually and scores the hits based on their proximity to hits from other genes (see Methods) (Table 2). The criterion for presuming that a cluster was “present” in either strain was that 50% of the genes in the MIBiG cluster were present [57]. The results indicate that 22 characterized clusters are present in both of our examined *A*. *flavus* morphotypes, consisting of the well-characterized aflatoxin gene cluster, as well as the aflavarin, aflatrem, and cyclopiazonic acid gene clusters. Nine clusters were predicted to be unique to S-morphotype AF70, including the agriculturally relevant carcinogen ochratoxin. To our knowledge, ochratoxin cluster genes have not been previously identified or characterized in *A*. *flavus* strains, thus AF70 may provide such an opportunity. Further study would be necessary to determine if even a minimal amount of ochratoxin production is detectable in AF70, and if not, then identify the cause for its non-production. As well, it may be important to determine if this cluster was inherited through horizontal gene transfer from a closely-related ochratoxigenic species such as *A*. *alliaceus* [58]. Six clusters are putatively unique to NRRL 3357.

**Table 2:** Percentage of genes identified as being present in *A*. *flavus* strains AF70 and NRRL 3357 that are putatively involved in the production of the indicated toxin*.

**Clusters in NRRL 3357 and AF70**				**Clusters in AF70**			
**Cluster Type**	**MIBiG ID**	**% in 3357**	**% in 70**	**Cluster Type**	**MIBiG ID**	**% in 3357**	**% in 70**
1,8-dihydroxynaphthalene	BGC0001258	100.0%	100.0%	Phytocassane / oryzalides	BGC0000672	45.5%	54.5%
4,4’-piperazine-2,5-diyldimethyl-bis-phenol	BGC0001234	100.0%	100.0%	Radicicol	BGC0000134	40.0%	80.0%
Aflavarin	BGC0001304	100.0%	100.0%	Alternapyrone	BGC0000012	40.0%	60.0%
Aspirochlorine	BGC0001123	100.0%	100.0%	Grayanic acid	BGC0001266	33.3%	66.7%
Ustiloxin B	BGC0000627	100.0%	100.0%	LL-Z1272 beta	BGC0001390	33.3%	66.7%
Aflatoxin	BGC0000007	100.0%	72.0%	Ochratoxin A	BGC0001030	33.3%	66.7%
Aflatrem	BGC0000629	94.1%	100.0%	Aphidicolin	BGC0000676	33.3%	50.0%
Aflatoxin/sterigmatocystin	BGC0000011	92.3%	73.1%	NG-391	BGC0001026	33.3%	50.0%
Sterigmatocystin	BGC0000152	88.2%	58.8%	Marneral	BGC0000669	25.0%	50.0%
Cyclopiazonic acid	BGC0000977	85.7%	100.0%				
Tenellin	BGC0001049	80.0%	80.0%	**Clusters in NRRL 3357**			
Chaetoglobosin	BGC0000968	71.4%	71.4%	**Cluster Type**	**MIBiG ID**	**% in 3357**	**% in 70**
Ferrichrome	BGC0000901	66.7%	66.7%	Acetylaszonal-enin	BGC0000293	66.7%	0.0%
Trans-resorcylide	BGC0001246	66.7%	66.7%	Aspyridone	BGC0000959	55.6%	33.3%
Depudecin	BGC0000046	66.7%	50.0%	Monodictyphen-one	BGC0000101	50.0%	0.0%
Paxilline	BGC0001082	62.5%	50.0%	T-toxin	BGC0000155	50.0%	0.0%
Desmethylbassianin	BGC0001136	60.0%	60.0%	Fujikurins	BGC0001305	50.0%	33.3%
Asperthecin	BGC0000684	57.1%	57.1%	Penitrem	BGC0001375	40.0%	0.0%
Dehydrocurvularin	BGC0000045	50.0%	50.0%				
Fumosorinone	BGC0001218	50.0%	50.0%				
PR toxin	BGC0000667	50.0%	50.0%				
Stipitatic acid	BGC0000154	50.0%	50.0%				

*Clusters with less than 50% genes present are presumed to be insufficient for production of the metabolite.

### Identification of high impact SNPs found in secondary metabolic gene clusters

An analysis of *A*. *flavus* strains AF70 and NRRL 3357 genomes allowed us to summarize and identify high impact SNPs. This analysis provides a foundation to describe the phenotypic differences between strains, such as differences in aflatoxin or sclerotium production. For this analysis, 13,403 the genomes were aligned, and SNPs were classified as having either high, moderate or low impact. High impact polymorphisms include exon deletions, premature stop codons and frameshift mutations. Moderate impacts consist of mutations occurring at the 3’ end of the gene, or mutations resulting in a change in amino acid. Low impact mutations are less likely to affect protein function. Our findings indicate that a majority of the genes (11,888) contain between 1 and 10 SNPs (Table 3) in NRRL 3357 The *A*. *flavus* S- (AF70) and L-strain (NRRL 3357) morphotypes differ in their production of aflatoxin (Fig 1). To examine if this is potentially due to structural differences between their aflatoxin clusters, we further examined those SNPs identified in their respective coding sequences. Table 4 and S2 Table detail the SNPs present in the aflatoxin gene clusters for both morphotypes examined.

**Table 3:** Summary of SNPs present in the *A*. *flavus* NRRL 3357 genome when queried against the *A*. *flavus* AF70 genome.

Impact	1 SNP	1<x<10 SNPs	>10 SNPs
High	987	349	0
Moderate	2,875	5,141	609
Low	2,762	6,398	908

**Table 4:** Genes in the aflatoxin biosynthetic cluster with SNP impacts classified as high, moderate, or low impact.

NRRL 3357 Gene	AF70 Gene	Gene (function)	High	Moderate	Low
AFLA_139430	AFLA70_210g001241	*aflU* (P450 monooxygenase)	4	6	8
AFLA_139250	AFLA70_106g003541	*aflL* (P450 monooxygenase)	1	16	60
AFLA_139420	AFLA70_210g001231	*aflT* (transmembrane protein)	1	8	8
AFLA_139140	AFLA70_106g003640	*aflYa* (NADH oxidase)	1	5	1
AFLA_139230	AFLA70_106g003561	*aflI* (cytochrome P450 monooxygenase)	0	22	26
AFLA_139220	AFLA70_106g003571	*aflO* (O-methyltransferase B)	0	18	53
AFLA_139240	AFLA70_106g003551	*aflLa (hypB*, putative oxygenase)	0	15	14
AFLA_139210	AFLA70_106g003581	*aflP* (O-methyltransferase)	0	11	19
AFLA_139150	AFLA70_106g003632	*aflY* (oxygenase)	0	11	9
AFLA_139410	AFLA70_210g001220	*aflC* (polyketide synthase A)	0	11	6
AFLA_139160	AFLA70_106g003561	*aflX* (monooxygenase)	0	10	23

**Fig 4:**
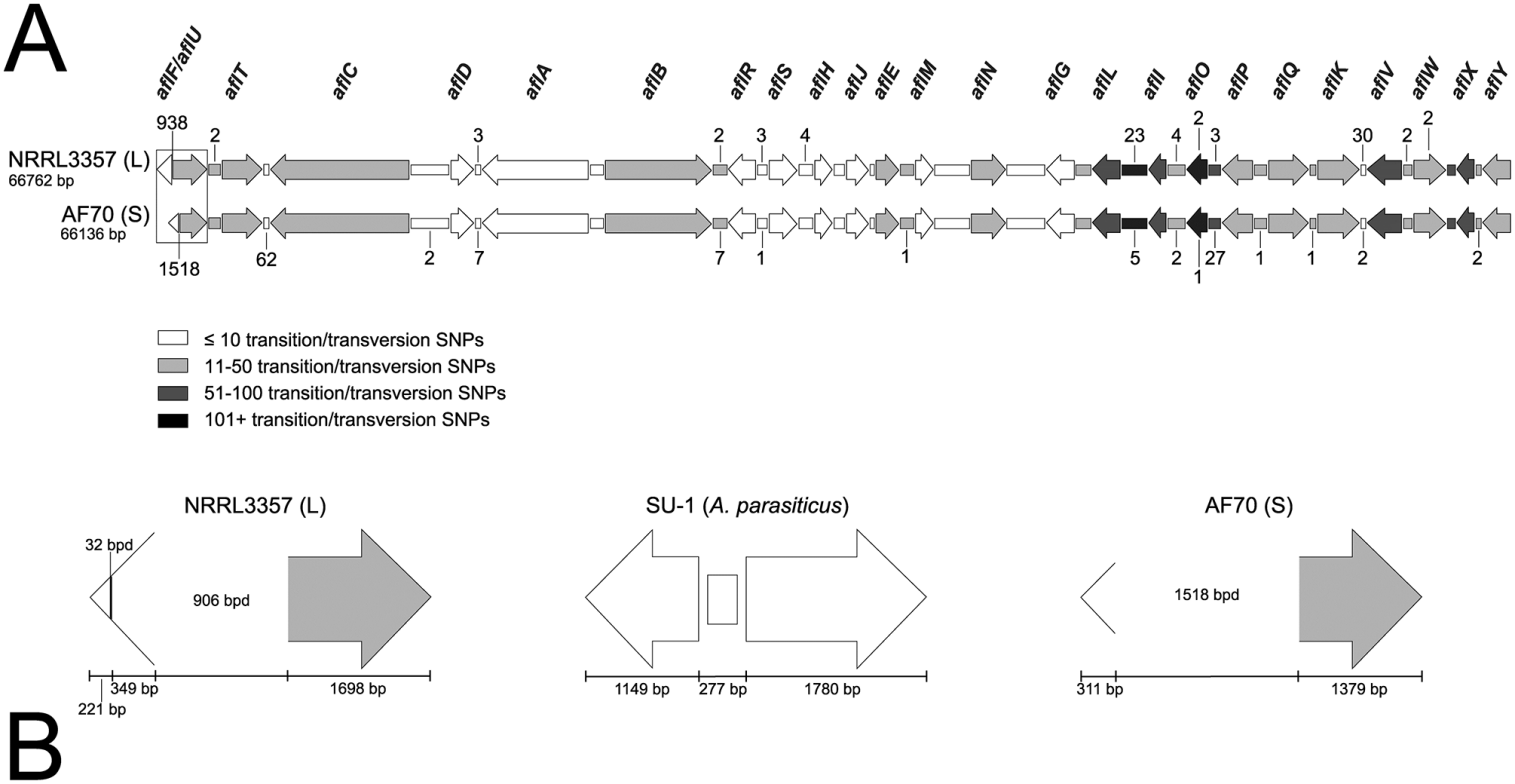
Schematic of the aflatoxin cluster as a comparison of polymorphisms that differentiate an *A*. *flavus* L-morphotype strain (NRRL 3357) from an *A*. *flavus* S-morphotype strain (AF70). The genes are shown as arrows and the intergenic regions are shown as boxes (A and B). Shading for each gene and intergenic region relates to the quantity of transition and transversion SNPs observed (legend). Any number noted above or below a gene or intergenic region for panel A represents the quantity of base pair deletions (bpd) found within the NRRL 3357 or AF70 cluster sequences, respectively. The boxed *aflF/aflU* regions in panel A are enlarged in panel B, for which the genes in this region (for both *A*. *flavus* morphotypes) are compared to the same (complete) genomic region in the SU-1 *A*. *parasiticus* strain. Areas noted with bpd indicate large-scale deletions observed.

To analyze the entire aflatoxin cluster, including intergenic regions, Fig 4 shows the results of our comparative polymorphism analysis between the aflatoxin gene clusters of NRRL 3357 and AF70, for which we found 1192 transition or transversion mutations (1.78% of the aflatoxin gene cluster) that differentiated the two morphotypes. The cluster of NRRL 3357 alone contained 116 deletions (0.17%), while the cluster of AF70 contained 742 deletions (1.11%). In total, more than 2000 SNPs within the aflatoxin cluster alignment could be used to differentiate these strains based on genotype. The greatest span of cluster alignment (9825 bp) having the lowest percentage of SNPs (0.35%) was found to encompass the start of the *aflC/aflD* intergenic region through the end of the *aflA/aflB* intergenic region. The second greatest span and second lowest percentage (7146 bp and 0.39%, respectively) encompassed the *aflR* gene through the end of the *aflJ/aflE* intergenic region. The intergenic region with the highest percentage (14.15%) of SNPs per span of genomic sequence (1039 bp) was *aflL/aflI*, and the gene with the highest percentage of SNPs (9.06%) per span of genomic sequence (1335 bp) was *aflO*. The highest concentration of deletions for both strains was found to reside in the *aflF/aflU* (*norB/cypA*) regions of their aflatoxin clusters. This region in L-morphotype NRRL 3357 exhibited 34 deleted bases not observed in AF70, and the S-morphotype AF70 exhibited 619 deleted bases not observed in NRRL 3357. Since both isolates are of the same mating type (*MAT1-1*), the presence of SNPs would be required to help differentiate the sequences, but their respective sequences were identical so our MLST analysis was based on five genomic loci.

It has been reported that the inability of *A*. *flavus* to produce G-aflatoxins is based on partial or complete deletion of the *aflU* gene, and that the amount of deletion observed in the *aflF/aflU* region will correlate with sclerotial morphology [59]. For example, the L-strain genotype for the *aflF/aflU* region results in an amplicon that is approximately 1 kb in size, while the S-strain genotype results in amplicon size of approximately 300 bp. Therefore, based on the report of Ehrlich and co-workers [59], NRRL 3357 is an L-morphotype strain and AF70 is an S-morphotype strain, which supported these strains being individuals and not sharing a VCG. Our MLST findings further corroborate the VCG results for these strains. VCGs in filamentous fungi are considered to be determined based on specific heterokaryon incompatibility (het) loci [60], and the identities of the relevant loci have not been identified in *A*. *flavus*.

In conclusion, the sequence and phenotypic differences quantified here between these *A*. *flavus* morphotypes are significant factors to consider in experimental design. In addition to being visibly different morphotypes, these model organisms produce significantly different levels of toxins. Here we describe additional differences in secondary metabolic gene cluster profiles, and identify several high impact SNPs within their aflatoxin gene clusters that could account for their differences in toxin production. This information should contribute to the further use and development of these strains in examining both fungal biology and their pathogenic potential.

## Supporting information

S1 Fig VCG assay for *A*. *flavus* strains AF70 (S-morphotype) and NRRL 3357 (L-morphotype). The results here illustrate a lack of cleft formation between mycelia of AF70 and NRRL 3357, containing complementary mutations, therefore indicating they are not vegetatively compatible.
(TIF)

S1 Table Gene Ontology enrichment. GO categories of genes that are unique to strains AF70 and NRRL 3357.
(XLSX)

S2 Table SNP analysis. Comprehensive list of SNPs identified in AF70 and NRRL 3357 aflatoxin gene cluster.
(XLSX)
