# Supplementary figures and images for: Whole genome comparison of Aspergillus flavus L-morphotype strain NRRL 3357 (type) and S-morphotype strain AF70
Source: PLoS One. 2018 Jul 2;13(7):e0199169. doi: 10.1371/journal.pone.0199169 (PMC6028093; doi:10.1371/journal.pone.0199169)

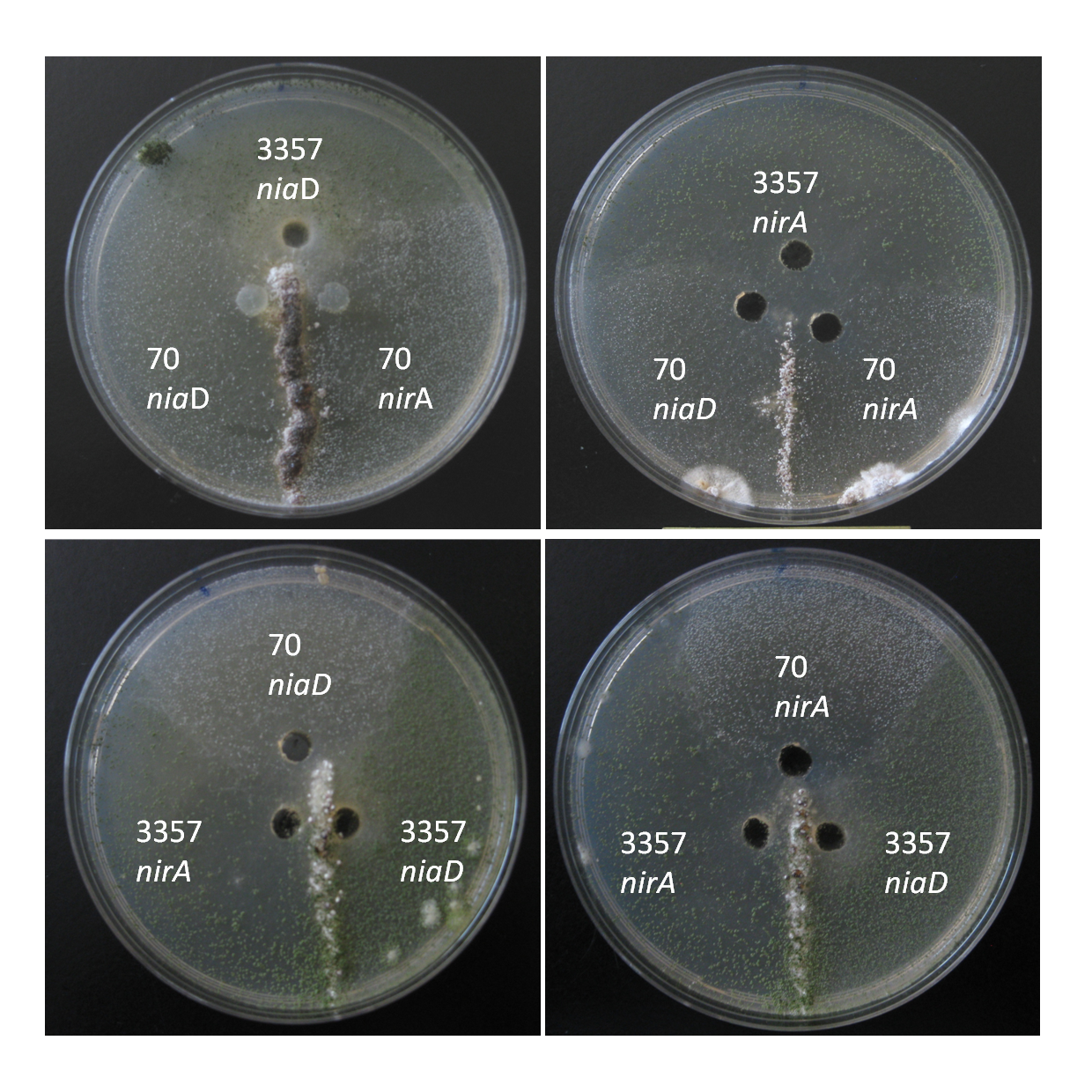

Supplement: S1 Fig — The results here illustrate a lack of cleft formation between mycelia of AF70 and NRRL 3357, containing complementary mutations, therefore indicating they are not vegetatively compatible. (TIF) [file pone.0199169.s001.tif]
